# Characterization of transcriptional modules related to fibrosing-NAFLD progression

**DOI:** 10.1038/s41598-017-05044-2

**Published:** 2017-07-06

**Authors:** Yi Lou, Guo-Yan Tian, Yu Song, Yin-Lan Liu, Yi-Dan Chen, Jun-Ping Shi, Jin Yang

**Affiliations:** 1 Center for Translational Medicine, The Affiliated Hospital of Hangzhou Normal University, Hangzhou, Zhejiang China; 2 Department of occupational medicine, Hangzhou Red Cross Hospital, Hangzhou, Zhejiang China

## Abstract

Based on the severity of liver fibrosis, low or high-risk profile of developing end-stage liver disease was present in nonalcoholic fatty liver disease (NAFLD). However, the mechanisms inducing transition from mild to advanced NAFLD are still elusive. We performed a system-level study on fibrosing-NAFLD by weighted gene co-expression network analysis (WGCNA) to identify significant modules in the network, and followed by functional and pathway enrichment analyses. Moreover, hub genes in the module were analyzed by network feature selection. As a result, fourteen distinct gene modules were identified, and seven modules showed significant associations with the status of NAFLD. Module preservation analysis confirmed that these modules can also be found in diverse independent datasets. After network feature analysis, the magenta module demonstrated a remarkably correlation with NAFLD fibrosis. The top hub genes with high connectivity or gene significance in the module were ultimately determined, including LUM, THBS2, FBN1 and EFEMP1. These genes were further verified in clinical samples. Finally, the potential regulators of magenta module were characterized. These findings highlighted a module and affiliated genes as playing important roles in the regulation of fibrosis in NAFLD, which may point to potential targets for therapeutic interventions.

## Introduction

Nonalcoholic fatty liver disease (NAFLD) represents a wide spectrum of disorders ranging from simple steatosis (nonalcoholic fatty liver, NAFL), nonalcoholic steatohepatitis (NASH), to cirrhosis or hepatocellular carcinoma (HCC). To date, NAFLD is one of the most common types of liver disease in the world^1^.

The diagnosis of NAFLD rests on clinicopathological criteria, requiring both clinical and biopsy-based information. The histological findings are graded as fatty changeand necroinflammatory using NAFLD activity score (NAS) scoring system, while fibrosis staging is useful to assess the severity and underlying cause of liver disease^2^.Challenges still lies in a lacking consensus for the classification of fatty liver disease, and absence of a uniform histological definition of NAFLD^3^.

Correspondingly, the phenotype and outcome of NAFLD is quite heterogeneous. For instance, though most NAFLD patients do not develop clinically significant hepatic disease, some patients can progress to cirrhosis, leading ultimately to HCC^4^. In addition, not all individuals with NASH finally develop cirrhosis or liver cancer^5^. On the contrary, although the severity of NASH generally correlates with the stage of fibrosis, some individuals with advanced fibrosis have relatively little NASH^6^. Moreover, the stage of fibrosis on liver biopsy independently associates with liver-related mortality^7^. When advanced fibrosis is present, absence of NASH is no longer prognostic. For these reasons,recent clinical data focus increasing attention on determining fibrosis, as it is a strong indicator of the risk extent for NAFLD^8^. Recently, our group have shown that fibrosis score is a useful predictor of long-term outcome in NAFLD patients^9^. Mechanisms leading to strongly differing progression of NAFLD, in term of fibrosis, have to be elucidated.

The high-throughput technology such as gene expression profiling has been applied to NAFLD and provides insights into molecular aspects of NAFLD progression^5^. However, gene lists based on differential expression analysis methods are biased against genes with large changes in expression, while lacking the consideration of the relationship between changing genes as a whole. In this sense, biological networks represent valuable tools for understanding system-level properties^10^. One network approach, named weighted gene co-expression network analysis (WGCNA) allows for the grouping functionally related genes into modules^11^.It is believed that modules are stable units since the overall function of a module can remain the same while individual gene expression can be changed or replaced by other genes with similar redundant functions^12^. Therefore, functional modules can more effectively reveal consistent differences during NAFLD progression.

In the present study, we applied a WGCNA approach to quantitatively assess the traits of mild or advanced fibrosing-NAFLD. Genome-scale modules of co-expressed genes with clear functional annotations were identified.

## Results

### Weighted co-expression network construction

**Figure 1:**
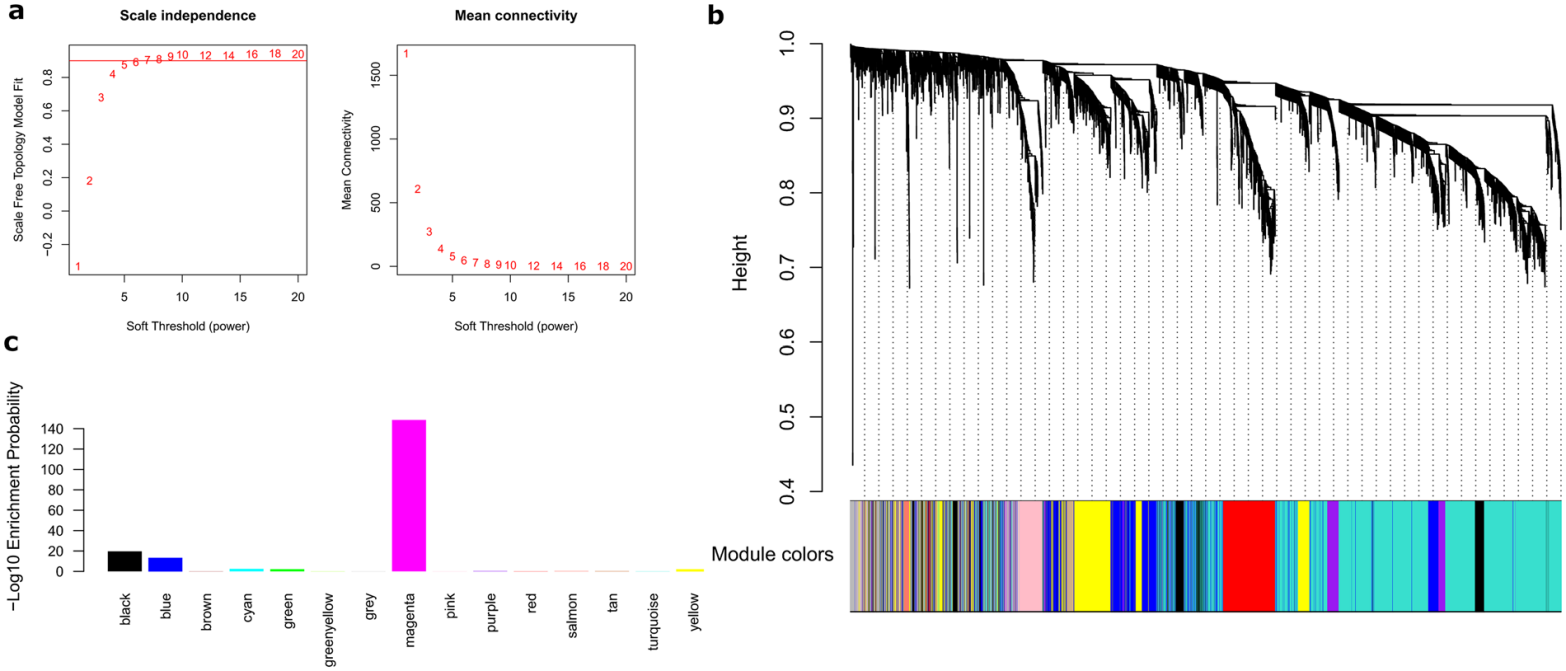
WGCNA network and module detection. (**a**) Selection of the soft-thresholding powers. The left panel shows the scale-free fit index versus soft-thresholding power. The right panel displays the mean connectivity versus soft-thresholding power. Power 5 was choosed, for which the fit index curve flattens out upon reaching a high value (>0.9). (**b**) Cluster dendrogram and module assignment for modules from WGCNA. Genes were clustered based on a dissimilarity measure (1-TOM). The branches correspond to modules of highly interconnected groups of genes. Colours in the horizontal bar represent the modules. 7012 transcripts were assigned to one of 15 modules including module grey. (**c**) Enrichment of DEGs in each module.

We used GSE49541 dataset in this study, since it contained the relatively largest NAFLD samples with clear fibrosis staging. After preprocessing the data, we applied the WGCNA package to compile the network. One outlier sample (GSM789152) was eliminated using hierarchical average linkage clustering (Supplementary File 1, Fig. S1). Keeping to the scale-free topology criterion, β = 5 was considered in this study (Fig. 1).

Following dynamic tree cut, the hierarchical clustering dendrogram identified 15 distinct gene modules, as shown in Fig. 1b. 348 genes failed to fit within a distinct group and were assigned to the grey module. The grey module was ignored in this study. The size of modules ranged from 38 (cyan module) to 2514 (turquoise module) genes. All attributes of genes and samples were shown in Supplementary File 2, Table S1–S2.

When compared with the mild samples, a total of 1134 differentially expressed genes (DEGs) were screened from the advanced NAFLD samples, including 762 upregulated and 371 downregulated genes. Consistent with the earlier research^13^, WGCNA modeling using DEGs could not match the scale-free feature of the network (Supplementary File 1, Fig. S2). DEG enrichment in each module was shown in Fig. 1c, in which DEG was mostly enriched in magenta module, and followed by black and blue module.

### Identification of meta-modules associated with NAFLD severity

**Figure 2:**
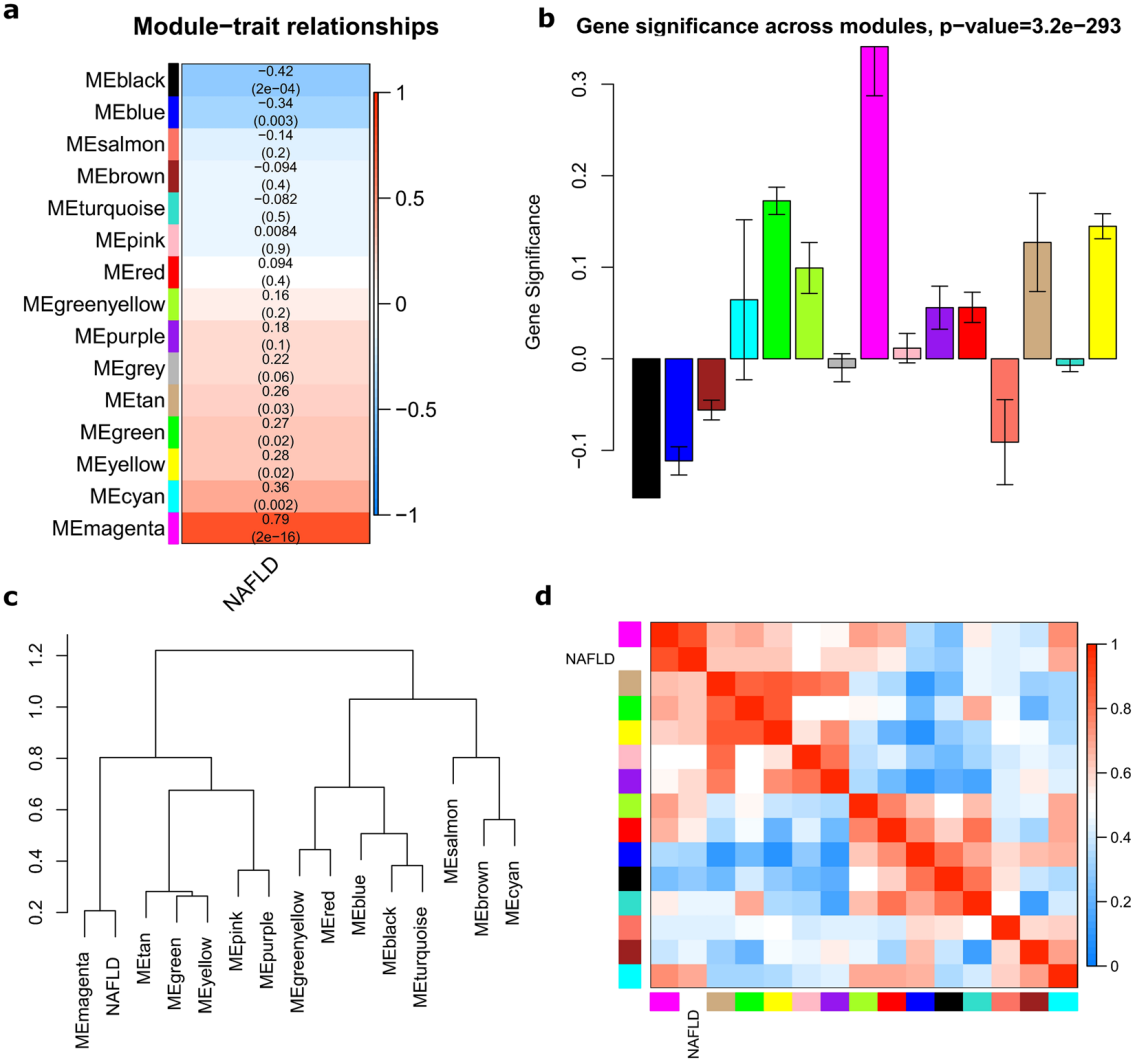
Module-trait and module-module associations of the network. (**a**) Each row corresponds to a module eigengene, column to a trait. Each cell contained the corresponding correlation and p value. The table was color-coded by correlation according to the color legend. The grey module included all the genes that can’t be clustered. (**b**) Module significance of each module, which is determined as the average absolute gene significance measure for all genes in a given module. (**c**,**d**) Eigengene network, including the clustering tree and heatmap, represents the relationships among the modules and the NAFLD trait. Meta-modules are defined as tight clusters of modules. The dendrogram indicates that magenta module and fibrosing-NAFLD trait are highly related. Conversely, blue and black modules are highly related, this meta-module is inversely correlated with fibrosis.

Next, we evaluated the relationship between each module and NAFLD status by correlating the eigengenes for each module with the fibrosis trait. Seven modules showed association evidence with p < 0.05(Fig. 2). Among them, five modules (tan, green, yellow, cyan, magenta) were positively correlated with fibrosis, thereafter named fibrosing-NAFLD modules. Two negatively correlated modules (blue, black) named NAFLD modules thereafter.

Module-module relationship, also called meta-module, is the groups of correlated eigengenes with correlation of eigengenes > 0.5. As shown in Fig. 2c,d, the dendrogram indicates that module mayanta is highly correlated with NAFLD fibrosis. While black and blue modules are highly related, their mutual correlations are stronger than their correlations with fibrosis trait.

### Stability and preservation of co-expression modules

To test the stability of the identified modules, internal analysis by repeating network construction and module identification on expression data that consists of resampled sets of the original dataset was performed^14^. The result proved the robustness of module assignments (Supplementary File 1, Fig. S3).

**Figure 3:**
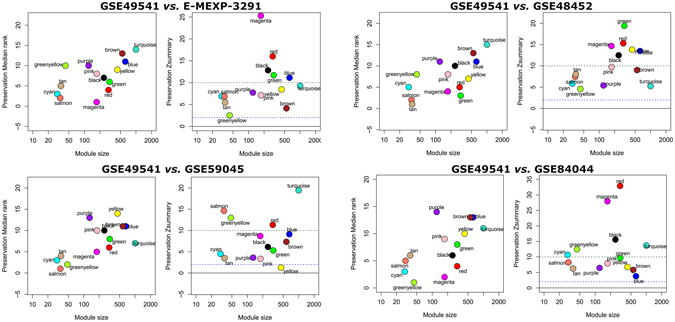
Preservation of GSE49541 network modules in different datasets. Each module is represented by its color-code and name. Left figure shows the composite statistic Preservation median rank. This measure tends to be independent from module size with high median ranks indicating low preservation. Right figure shows preservation Zsummary statistic. The dashed blue and green lines indicate the thresholds Z = 2 and Z = 10, respectively. Zsummary < 2 implies no evidence for module preservation, 2 < Zsummary < 10 implies weak to moderate evidence, and Zsummary > 10 implies strong evidence for module preservation. Fibrosing-NAFLD modules (tan, green, yellow, cyan, magenta) show high preservation statistics summary than expected by random chance using bootsrapping validation procedures.

To ask if the identified modules were common in different datasets, an independent validation was performed. We retrieved 8 datasets relevant to NAFLD. All samples were from human liver tissue. Fibrosing-NAFLD modules (tan, green, yellow, cyan, magenta) were stable across E-MEXP-3291, GSE48452, and GSE59045. To examine if these identified modules were also presented in fibrosing liver disease such as HBV, GSE84044 dataset containing 124 samples with different stages of fibrosis was used as a specific control (Fig. 3).

In addition, NAFLD modules (blue, black) were preserved in other 5 datasets (GSE17470, GSE24807, GSE37031, GSE46300, and GSE63067) according to the summary preservation statistics, while fibrosing-NAFLD modules showed weak to none evidence for module preservation (Supplementary File 1, Fig. S4).

### Functional enrichment analysis of the gene modules of interest

Gene ontology annotation and enrichment analysis were accomplished using DAVID version 6.8 (https://david-d.ncifcrf.gov/)^15^. Top biological processes and KEGG pathway in each module was shown in Table 1.

**Table 1:** Top GO and pathway enrichment in each module.

	module	Category	Term^a^	PValue	FDR
Fibrosing-NAFLD module	magenta	GOTERM_BP	GO:0030198~extracellular matrix organization	1.51E-13	2.50E-10
	cyan	GOTERM_BP	GO:0097284~hepatocyte apoptotic process	2.45E-02	2.73E + 01
	yellow	GOTERM_BP	GO:0043687~post-translational protein modification	1.29E-06	2.21E-03
	green	GOTERM_BP	GO:0032486~Rap protein signal transduction	6.16E-04	1.01E + 00
	tan	GOTERM_BP	GO:0050790~regulation of catalytic activity	7.24E-03	9.18E + 00
NAFLD module	black	GOTERM_BP	GO:0022904~respiratory electron transport chain	6.68E-08	1.09E-04
	blue	GOTERM_BP	GO:0044281~small molecule metabolic process	6.21E-39	1.10E-35
	module	Category	Term	PValue	FDR
Fibrosing-NAFLD module	magenta	KEGG_PATHWAY	hsa04510:Focal adhesion	2.37E-11	2.91E-08
	cyan	KEGG_PATHWAY	hsa03030:DNA replication	8.05E-02	5.08E + 01
	yellow	KEGG_PATHWAY	hsa04120:Ubiquitin mediated proteolysis	8.90E-04	1.13E + 00
	green	KEGG_PATHWAY	hsa04720:Long-term potentiation	1.73E-02	1.94E + 01
	tan	KEGG_PATHWAY	hsa04015:Rap1 signaling pathway	5.78E-02	4.58E + 01
NAFLD module	black	KEGG_PATHWAY	hsa04932:Non-alcoholic fatty liver disease (NAFLD)	3.33E-06	4.19E-03
	blue	KEGG_PATHWAY	hsa01100:Metabolic pathways	5.96E-18	7.81E-15

Globally, top 5 biological processes were enriched in the modules of interest, including small molecule metabolic process (blue, FDR = 1.10E-35), extracellular matrix organization (magenta, FDR = 2.50E-10), gluconeogenesis (blue, FDR = 2.70E-06), extracellular matrix disassembly (magenta, FDR = 7.31E-06), respiratory electron transport chain (black, FDR = 1.30E-04). Top 5 enriched pathways were as follows: hsa01100: Metabolic pathways (FDR = 7.81E-15) in the blue module, hsa01200: Carbon metabolism (FDR = 8.11E-11) in the blue module, hsa04510: Focal adhesion (FDR = 2.91E-08) in the magenta module, hsa04512: ECM-receptor interaction (FDR = 1.91E-06) in the magenta module, and hsa04932: Non-alcoholic fatty liver disease (NAFLD)(FDR = 4.19E-03) in the black module. The complete annotation for each module was provided in Supplementary File 2, Table S3–S4.

### Network analysis of the gene modules of interest

After viewing the global properties of the interesting networks, we next examined the gene constitution of particular modules based on network unique properties, such as gene significance (GS), module membership (MM) and intramodular connectivity (K.in).

Abstractly speaking, a gene is more meaningful with high GS, MM and K.in^16^. Thus, if MM, K.in or GS of the specific module were significantly connected and associated with the NAFLD fibrosis status, it implied that the module serves an more important biological role to NAFLD progression.

**Figure 4:**
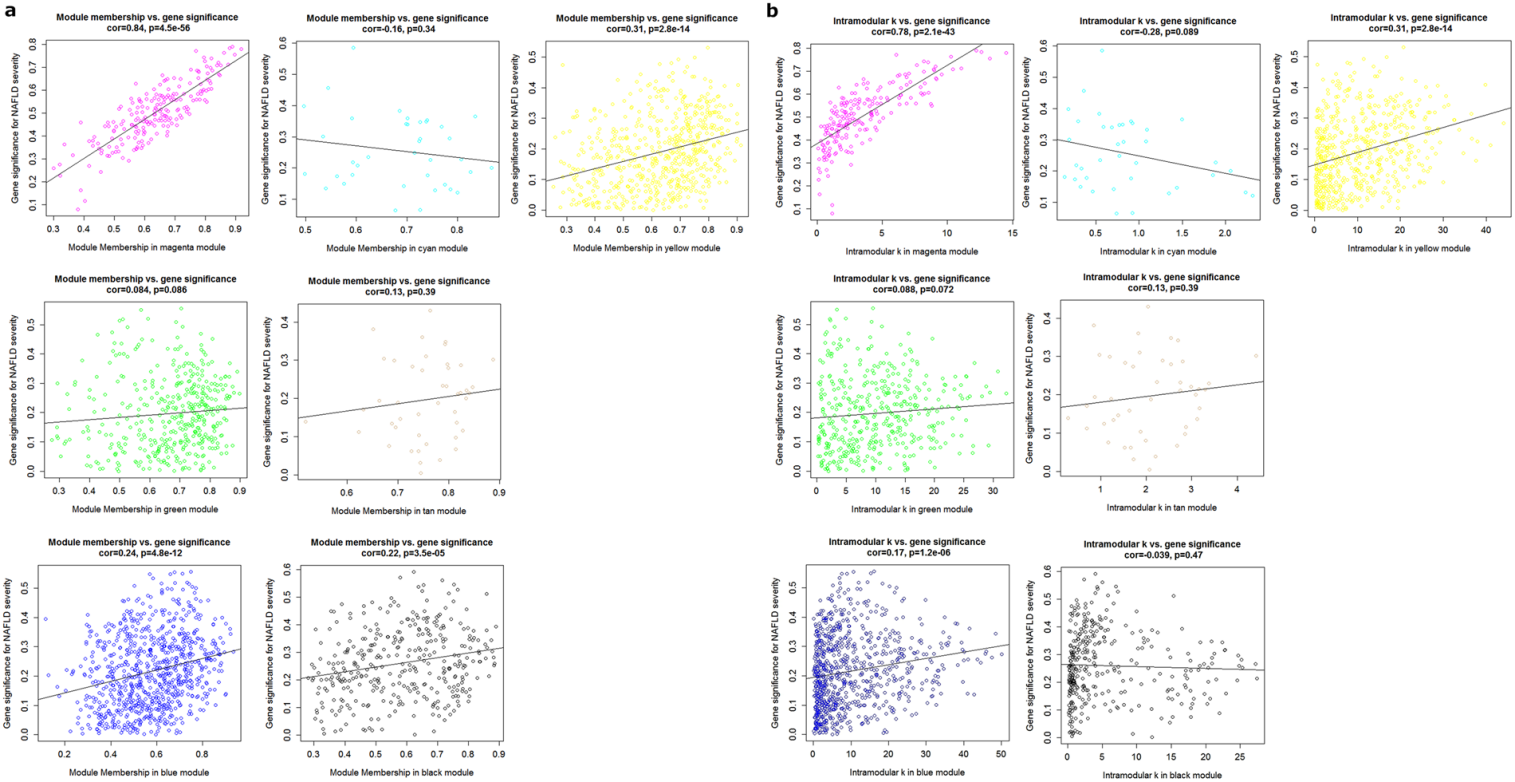
Module features of GS, MM and K.in. (**a**) Modules significantly correlated with NAFLD status (mild versus advanced fibrosis). Each point represents an individual gene within each module, which are plotted by GS on the y-axis and MM on the x-axis. The regression line, correlation value and p-value are shown for each plot. (**b**) Correlation of the K.in (x-axis) and the GS (y-axis).

Of the seven interesting modules, significant correlations were observed between MM and GS in the yellow, blue, black, and magenta modules. We also found a markedly correlation between GS and K.in in the yellow, blue, black, and magenta modules (Fig. 4). Overall, module magenta shows the best as reflected by its strongly positive correlations(r = 0.84, p = 4.5E-56 in GS vs.MM; r = 0.78, p = 2.1E-43 in GS vs.K.in). These results indicated that magenta module is heavily involved in NAFLD fibrosis progression.

### Characterization of the magenta module content and hub genes

**Figure 5:**
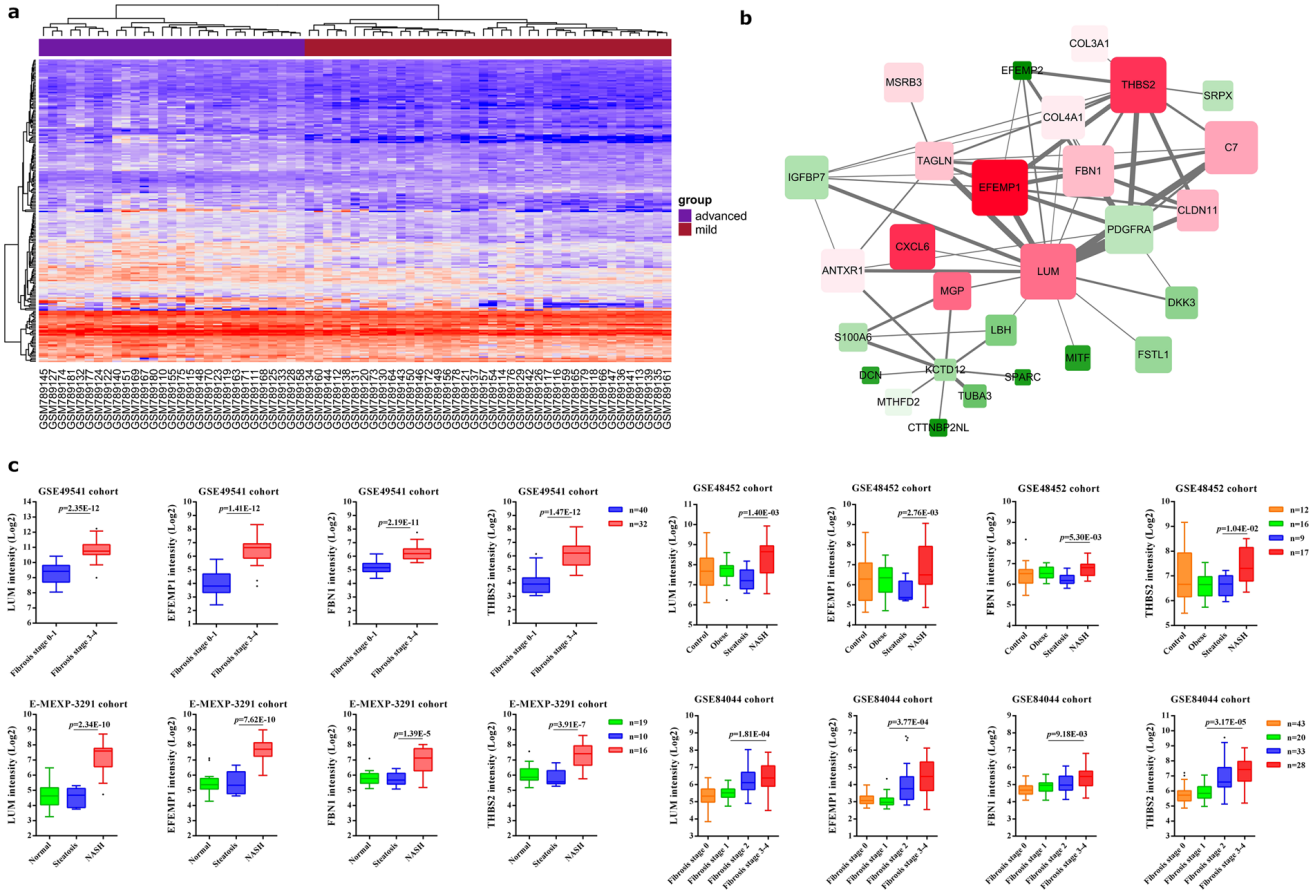
Characterization of the magenta module. (**a**) Gene expression heat-map of module magenta. (**b**) Interaction of gene co-expression patterns in the magenta module. The module was visualized using Cytoscape 3.0 software. The node colors coded from green to red (low to high) indicate the fold change when compared mild with advanced NAFLD state. The node size is proportional to the significance of the expression changes compared to mild NAFLD. (**c**) Four hub genes expression pattern in liver tissues according to GSE49541, E-MEXP-3291, GSE48452 and GSE84044 cohort. Data were shown as box and whisker plot. Limma package was used for statistical analysis.

A network view of magenta module, modeled by cytoscpae with TOM ≥ 0.1, is depicted in Fig. 5. The K.in count for each gene ranged from 0.1 to 14.5, with an average of 3.49 ± 2.90. The GS score for each gene ranged from −0.58 to 0.79, with an average of 0.34 ± 0.03. The MM for each gene ranged from −0.68 to 0.92, with an average of 0.43 ± 0.03. Using STRING^17^ or GeneMANIA^18^ database to model the network gave the similar results (Supplementary File1 Fig. S6).

Focusing on the magenta module, we explored those core genes that had a high significance for NAFLD status, as well as high K.in. Network top interesting genes of the magenta module based on the above two indexes are listed in Table 2. The three top network hub genes (LUM, FBN1, and THBS2) based on K.in and the three top genes (EFEMP1, THBS2, and LUM) ranked on GS were disclosed.

**Table 2:** Hub genes in module magenta.

	Gene	GS	GSRank	k.in	k.in Rank	Potential Transcription factor
Network Hub genes (based on k.in)	LUM	0.779	3	14.511	1	ESR1, BACH1, TRIM28, RUNX2
	FBN1	0.756	6	13.234	2	TP63
	THBS2	0.785	2	12.597	3	ESR1, TP53, BACH1, TP63, RELA
Network top genes (ranked on GS)	EFEMP1	0.790	1	12.224	4	ZNF217, BACH1, TP63
	THBS2	0.785	2	12.597	3	ESR1, TP53, BACH1, TP63, RELA
	LUM	0.779	3	14.511	1	ESR1, BACH1, TRIM28, RUNX2

Specifically, several proteomic studies have identified lumican (LUM) is expressed differentially across the progressive stages of NAFLD^19^. Upregulated expression of LUM is in association to hepaticfibrosis^20^. Moreover, in animal studies, LUM is a prerequisite for hepatic fibrosis, which involves collagen fibrillogenesis, and matrix turnover^21^.

To the best of our knowledge, there was nothing directly implicating EFEMP1, FBN1 and THBS2 reported to be associated with severe NAFLD. However, EFEMP1 has been showed decreased expression in HCC tissue^22^. The following two genes, FBN1 and THBS2, were belong to the cellular adhesion and extracellular matrixconstituent. FBN1, has been shown mounting a hepatic progenitor cell response for tissue repair in rat liver^23^. THBS2 was found over-represented in patients with vascular liver lesions such as sinusoidal dilatations^24^.

All these four genes were significantly upregulated in advanced fibrosing-NAFLD (GSE49541). High expression of these genes was also confirmed in advanced NAFLD in other cohorts (E-MEXP-2191, GSE48452 and GSE84044, Fig. 5c). Conversely, these genes were not differentially expressed in the NAFLD dominant datasets (Supplementary File 1, Fig. S5), suggesting EFEMP1, FBN1 and THBS2 maybe the novel candidate biomarkers for fibrosing-NAFLD.

### Hub genes were significantly up-regulated in the livers from NAFLD patients and mices

To investigate if hub genes were modified in fibrosising-NAFLD, the production of these genes were further examined in livers from NAFLD animals and patients.

**Figure 6:**
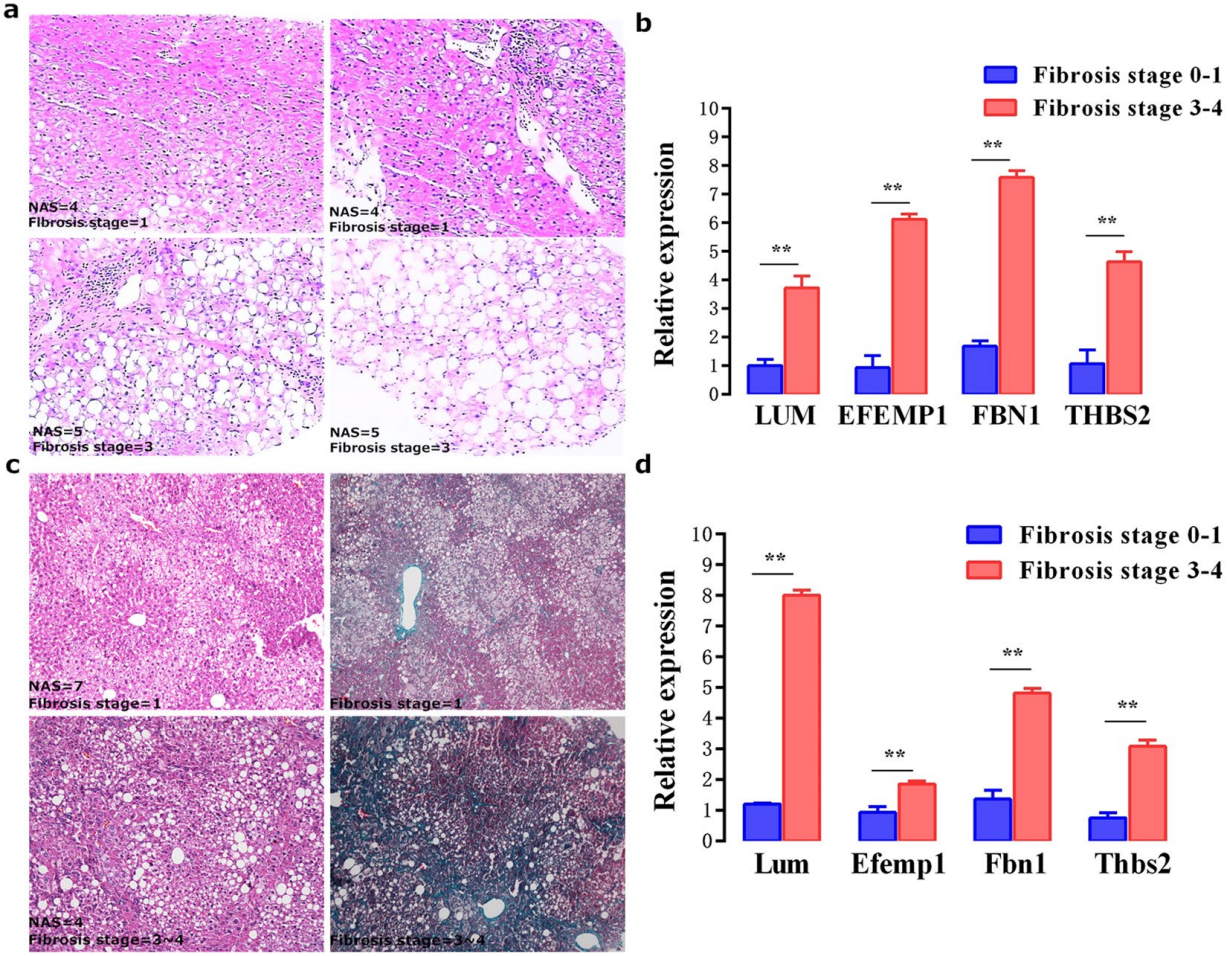
Expression of hub genes in different fibrosis stages of NAFLD. (**a,b**) The representative HE staining of NAFLD patients with different fibrosis stages were shown. Quantification of hub genes was presented. (**c,d**) Liver sections were stained with HE in mice fed with HFHC diet at 20 weeks. Masson’s trichrome staining was used to detect the accumulated collagen. The hepatic production of hub genes was confirmed and presented. **P < 0.01.

Liver biopsy tissues from NAFLD patients were stained to assess disease severity. Compared with NAFLD fibrosis stage 0–1 patients (n = 4), LUM, EFEMP1, FBN1, and THBS2 were remarkablely up-regulated in the fibrosis stage 3–4 state patients (n = 4) (Fig. 6a,b).

ApoE^−/−^ mice receiving a HFHC diet are the well-established animal models mimicking human NAFLD^25^. As expected, ApoE^−/−^ mice fed a HFHC diet for 20 weeks evidenced hepatic steatosis, ballooning, hepatic inflammation and increased fibrosis. Similarly, Lum, Efemp1, Fbn1, and Thbs2 were significantly higher in livers from NAFLD mice with severe fibrosis compared to livers from mice with mild fibrosis (n = 3 in each group, Fig. 6c,d). The data *in vivo* above suggests a close relationship between hub genes and NAFLD fibrosis.

### Functional organization of the magenta module

Next, function relevance of magenta module was reannotated using DAVID tool. With the cutoff set as FDR <0.1, focal adhesion, ECM-receptor interaction, and phosphatidylinositol 3-kinase(PI3K)-Akt signaling pathway constitue the main pathways in magenta module. In complete accord with the phenotype, GOterm extracellular matrix organization and cell adhesion were significantly enriched.

**Figure 7:**
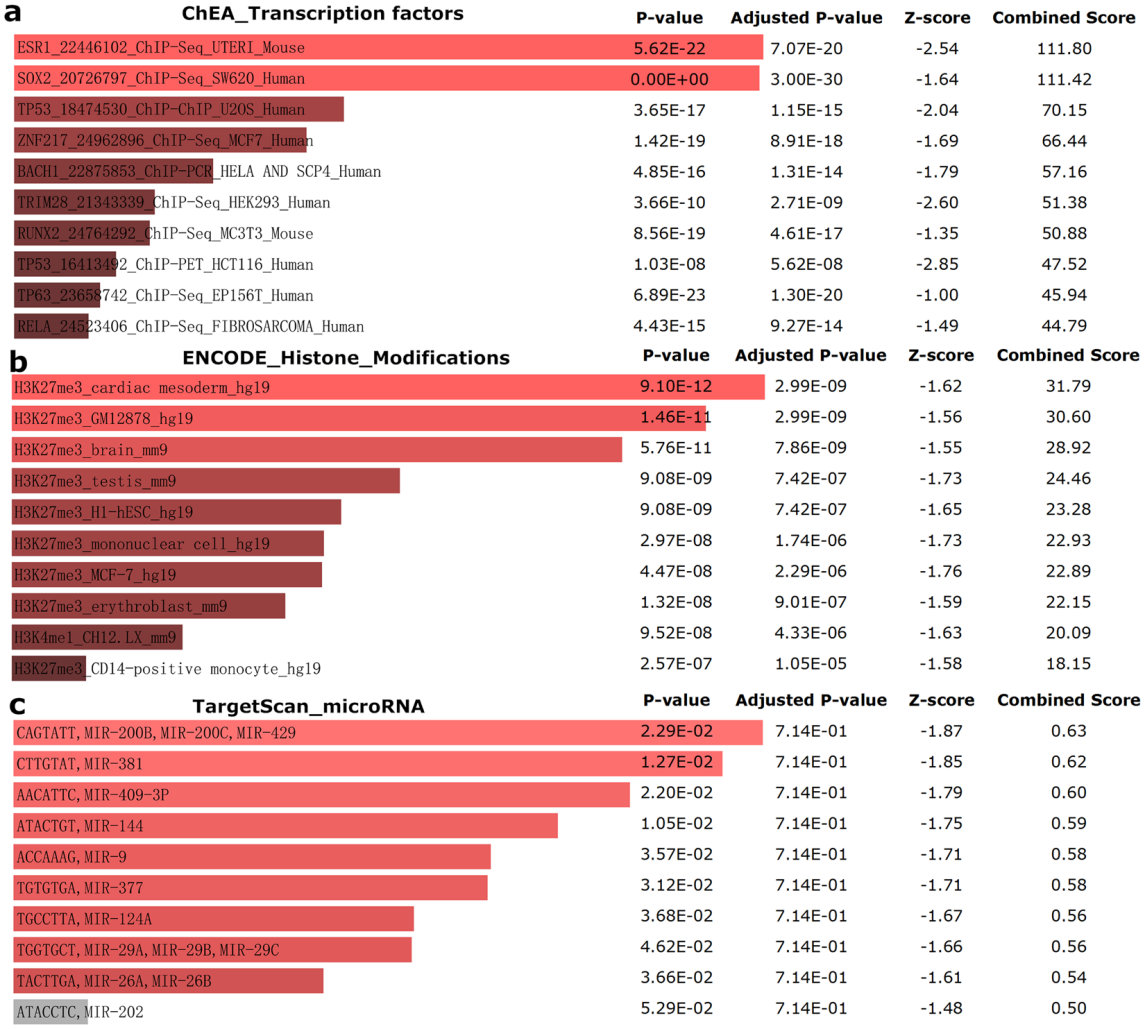
Potential factors regulating genes in magenta module. (**a**) Transcription factors. (**b**) Histone modification markers. (**c**) Enriched seed and its associated microRNA.

Since co-expressed genes may be co-regulated by the common transcription factors (TFs) and microRNAs, we performed gene-set enrichment analysis using ChEA, Encode, and Targetscan database^26–28^ for magenta module. Significant enrichments of transcription factors were observed for ESR1, SOX2, TP53, etc (Fig. 7; Supplementary File 2, Table S5–S7). Several TFs were reported to be functionally associated with NAFLD. For instance, CpG island methylation of ESR1 was found to be involved in lipid and glucose metabolism, and the progression of fibrosis in mouse feeding with methyl-deficient diets^29^. SOX2 expression may predict the prognosis of HCC patients^30^. As a metabolic modulator, growing evidences highlight TP53 a new player in NAFLD pathogenesis^31^. BACH1 gene ablation reduces steatohepatitis in mouse^32^. Specifically, RELA (p65), is well-known for its trigging inflammatory responses in NAFLD^33^. RUNX2 downregulation is involved in cirrhotic liver^34^. Most recently, TRIM28 was found to modulate the prevalence of obesity in the population^35^. The other two genes (ZNF217, TP63) enriched in magenta module have not been reported for their relationship to liver.

H3 lysine 27 trimethylation (H3K27me3) was strongly enriched for most of the genes in module magenta. Recent paper has shown that in human NAFLD-associated HCC, level of H3K27me3 was correlated positively in tumors compared with nontumor tissues^36^.

Finally, the most enriched miRNAs were observed for miR-200b, miR-200c and miR-429. In high fat diet feeding rats, miR-200a, miR-200b and miR-429 were significantly correlated with a severity of NAFLD-specific liver pathomorphological features^37^. Another study has showed, miR-200a, miR-200b, and miR-200c were up-regulated in NAFLD^38^.

## Discussion

Here, we present a systematic WGCNA of NAFLD with either mild fibrosis or advanced fibrosis. Among the 14 modules discovered in this study, seven modules were significantly associated with disease progression.

As an alternative to traditional differential expression analyses which centered on individual genes distinguishing the status of NAFLD, in an unbiased manner, WGCNA groups co-expressed genes that are biologically integrated on a genome-wide scale. We found several features in term of the relationship between WGCNA and DEG analysis. First, using differential expression genes to model WGCNA network is not suggested, since it completely invalidates the scale-free topology assumption. Second, we found that, globally, DEG genes have less connectivity than non-DEG genes (9.57 ± 12.91 vs. 24.93 ± 37.59, respectively), and this association does not follow a simple monotonic trend in each module. Third, DEG genes tend not to be hub genes as determined by connectivity in most modules. However, DEG numbers show a significant enrichment in seven important modules (Fig. 1c). Thus, WGCNA and DEG showed efficient mutual complementation when for transcriptome analysis.

Results suggest that the modules identified here are biologically rational. First, fibrosing-NAFLD and NAFLD modules were clearly separated by WGCNA approach. Module preservation was extensively studied among different datasets. Second, most of the identified modules are enriched for specific GO terms and KEGG pathways. For instance, module black, together with module blue, are markedly inverse correlated with NAFLD fibrosis. Both the black and blue modules were enriched in respiratory electron transport chain and small molecule metabolic process, which have already been implicated in oxidative stress, and mitochondrial dysfunction for NAFLD^39^. KEGG analysis showed that these modules are directly related to NAFLD. Moreover, hub genes in these modules are implicated in NAFLD as reported by literature annotation. As the hub gene of blue module,selenium-binding protein 1(SELENBP1) has been shown to be downregulated in the liver tissue of HCC patients and the association of its gradual loss with an increased malignant grade^40^. Thus, it is temping to speculate that the NAFLD modules (black, and blue) dominate the period of NAFLD when fibronesis is not obvious.

Recently, the systems biology approach for NAFLD has been applied in studies by integrating genomic data and metabolic networks. One study showed that metabolism of amino acids, chondroitin and heparan sulphates seem to be involved in the appearance of NASH^41^. In our analysis, we observed regulation of cellular amino acid metabolic process in black or blue module, including branched-chain, sulfur, alpha-amino, serine family, aspartate family amino acid metabolic process. Simultaneously, cellular response to amino acid stimulus was also enriched in magenta module with relatively small propability, which suggests the continuous amino acid metabolic stress during NAFLD pathogenesis.

This study highlights the importance of magenta moduleas as a driver of fibrosis based on the meta-module, and further through the network feature (GS, MM and K.in) analysis. Enriched GO terms or pathway are highly concordant. In particular, extracellular matrix (ECM) and PI3K pathway were top core gene sets of the magenta module. The ECM is mainly composed of an intricate interlocking mesh of fibrillar and non-fibrillar collagens, elastic fibers and glycoproteins^42^, which is a highly dynamic structure undergoing controlled remodelling^43^. The deposition of increased and abnormal ECM is the hallmark of liver fibrosis^44^. Correspondingly, the PI3K signaling pathway has been shown to regulate procedures associated with hepatic stellate cell (HSC) activation such as collagen synthesis and cell proliferation^45^. Inhibition of PI3K signaling in HSCs suppresses ECM deposition, type I collagen synthesis, and reduce the expression of profibrogenic factors. In a feed-back manner, collagen crosslinking increases stiffness, β1 integrin clustering, PI3K signalling and focal adhesion formation to drive the disease progression^43^.

The progression of NAFLD from mild steatosis up to severe steatohepatitis and even cirrhosis or HCC, varies widely between individual patients. Recently, one study was to assess the histological severity of NAFLD in a cohort with serial biopsy data, and contrary to current dogma, this study suggests that steatosis can directly progress to NASH and clinically significant fibrosis^46^. Since causality between liver fibrosis and the prognosis of the liver disease is well recognized, the intervening measure may have to be adjusted in different subsets of NAFLD patients.

To demonstrate the usefulness of our modules in the development of efficient NAFLD interfere strategies, given magenta module as an example, small compounds derived from the Library of Integrated Network-based Cellular Signatures (LINCS) L1000 platform^47^ affecting the gene expression was provided in Supplementary File 2 Table S8–9. Next, novel potential biomarkers including EFEMP1, THBS1, FBN1 were disclosed in magenta module, after extensive cross-validation. Another noteworthy was the link between the regulators (transcription factors and epigenetic markers) and the co-expression mode of genes in magenta module, which may suggest the regulatory circuit during NAFLD progression. In the future, more experiments are needed to validate these discovery clues.

In summary, this study generated a comprehensive and unbiased snap-shot of the modules as well as genes in fibrosing-NAFLD. In particular, magenta module and genes regulating ECM remodelling during NAFLD progression deserve further attention. An identification of mechanistically linked key module and regulators will aid intervention development.

## Methods

### Gene expression dataset and processing

Transcription profile of NAFLD was downloaded from the Gene Expression Omnibus (GEO) with accession number GSE49541. The raw data were corrected and normalized using the RMA function of affy package of R 3.2.0 in Bioconductor. This datatset represents two clinically defined pathological groups at the extremes of NAFLD: mild NAFLD (n = 40, fibrosis stages 0–1), with little risk of developing severe liver disease; advanced NAFLD (n = 32, fibrosis stage 3–4), with significant likelihood of developing liver-related morbidity and mortality. As described earlier, the two groups were matched for gender, age and body mass index^48^. Differential expressed genes (DEG) were considered by using the criterion with Benjamini & Hochberg adjusted p value less than 0.05.

The microarray datasets referenced during the study (E-MEXP-3291, GSE45428, GSE50594 and GSE84044, etc) are available in a public repository from EBI (http://www.ebi.ac.uk/) or NCBI GEO. All the other data supporting the findings of this study are available within the article and its Supplementary File 1 Table S1. It is important to emphasize we include only the human NAFLD samples in our study. Full experimental methods and detailed descriptions of these public data sets can be found in the original references.

### WGCNA network construction and module detection

After normalization, we removed lowly and nonexpressed genes by selecting probes with a mean expression in the top 50% of all probes. Next, genes with expression variance above average level were selected. Different probes targeting the same gene were collapsed. These steps finally resulted in 7012 genes to infer co-expression networks.

Networks were formed following the protocols of WGCNA^49^. A pairwise pearson correlation coefficient matrix was first computed, and an adjacency matrix and topological overlap matrix (TOM) were constructed^11^. TOM is a parameter referring to the interconnection between two genes, and a module is a cluster of genes with high topological overlap. Modules were identified on the dendrogram using the dynamic tree cut algorithm^50^. The module eigengene (ME) is defined as the first principal component of a given module, which can be considered a representative of the gene expression profiles in a module. Module Membership (MM), also known as eigengene-based connectivity (kME), is defined as the correlation between the module eigengene and gene expression values. Genes weakly correlated with all of the MEs (|kME| < 0.7) were assigned to none of the modules. Finally, the interesting module network was visualized by Cytoscape^51^.

Module preservation implemented in WGCNA was used to detect the conservation of gene pairs between two networks^52^. Briefly, three types of network based module preservation statistics, including density based preservation statistics, connectivity based preservation statistics, and network based statistics have been identified. Then, two composite measures have been defined. Median rank is defined as the mean of median ranks computed for connectivity and density measures of each module. Zsummary is used to assess the significance of observed statistics and is defined as the mean of Z scores computed for density and connectivity measures. We utilize median rank to identify module preservation and Zsummary to assess significance of module preservation via permutation testing 200 times.

### Feature vectors in WGCNA network

Gene significance (GS) was defined as the the correlation between individual genes and NAFLD trait. The intramodular connectivity (K.in) was calculated as the summation of adjacency performed over all genes in a particular network. If GS and MM are highly correlated, it means that genes are the most important elements of modules and are highly significantly associated with the trait. Generally, the MM is high in relation to k.in, and a higher correlation indicates that a gene is more important to the given module^11^.

Hub genes tends to be located in the centre of a network, highly connected with other genes and hence of high functional significance. Therefore, a gene with high GS, high MM and high K.in in a module was considered to be a hub gene.

### Functional annotation of the modules

Gene ontology (GO) and KEGG pathwayenrichment analysis for network modules were performed using the Database for Annotation, Visualization and Integrated Discovery (DAVID)^15^. In DAVID, an overrepresentation of a term is defined as a modified Fisher’s exact P value with an adjustment for multiple tests using Benjamini method.

In addition, we related interesting modules to biological curated gene sets on the basis of Enrichr (http://amp.pharm.mssm.edu/Enrichr), which currently contains a large collection of diverse gene set libraries available for analysis^53^. In Enrichr, apart from the Fisher exact test used in most gene list enrichment analyses, a z-score test statistics capture the deviation from the expected rank by the Fisher exact test. Then, a combined score evaluating the enrichment was computed as follows: C = log(p)⋅Z. Where C indicates the combined score, p is the p-value computed using the Fisher exact test, and Z is the z-score computed by assessing the deviation from the expected rank.

### Ethical considerations

All the experiments protocols involving humans and animals were approved by the Human Ethics Committee of the affiliated hospital of Hangzhou Normal University. Methods were carried out in accordance with the approved guidelines and regulation. Written informed consent was obtained from all participants. Appropriate care was given to all animals included for experiments.

### Patients

The criteria for NAFLD were based on those recommended by the Chinese Liver Disease Association. Liver biopsies from NAFLD patients with different stages of fibrosis, identified between Feb 2013 and Oct 2016 in the Department of Liver Diseases, affiliated hospital of Hangzhou Normal University, China was collected and stored at −80 °C before analysis. Paraffin embedded liver tissues were used for immunohistochemistry (IHC).

### Animal studies

Four-week-old male ApoE^−/−^ mices were purchased from Model Animal Research Center of Nanjing University (Nanjing, China). All mices were bred in a specific pathogen-free facility and maintained in a 12-hour light-dark-cycle at room temperature and fed ad libitum. The mices were divided into two groups and allocated into either a normal Chow-diet (Normal group) or high fat high cholesterol-diet (HFHC group). HFHC diet was from Research Diets, New Brunswick (D12079B; Research Diets New Brunswick, NJ). At the end of the experiments, a part of the liver tissue was fixed with 10% formaldehyde and the remaining liver was snap frozen.

### Liver histology

Formalin-fixed liver tissue was processed into 4 μm thick paraffin sections and stained with hematoxylin and eosin (HE) and Masson’s staining. Degree of NAFLD activity score (NAS) were scored according to NASH clinical research network (CRN) scoring system.

### Quantitative real-time PCR

Hepatic mRNA levels were analyzed by qRT-PCR using a 7900 Real Time PCR System (Applied Biosystems, USA). The RNA was isolated with TRIzol (Invitrogen, USA). cDNA was synthesized using 2 μg of total RNA with PrimeScript™ Reverse Transcriptase (Takara). Amplification reactions were performed using the SYBR® Premix Ex Taq kit (Takara) and 0.2 µM of gene specific primers (Supplementary File 1 Table S2) and PCR products were verified by melting curve analysis. The relative quantification expression was calculated using the delta-delta Ct method with each gene normalized to GAPDH.

## Electronic supplementary material

Supplementary file 1

Supplementary file 2
