# Supplementary material for: Characterization of transcriptional modules related to fibrosing-NAFLD progression
Source: Sci Rep. 2017 Jul 6;7:4748. doi: 10.1038/s41598-017-05044-2 (PMC5500537; doi:10.1038/s41598-017-05044-2)
Supplement: Supplementary file 1 [file 41598_2017_5044_MOESM1_ESM.pdf]

Characterization of transcriptional modules related to fibrosing-NAFLD progression

Yi Lou [1, 2], Guo-Yan Tian [1], Yu Song [1], Yin-Lan Liu [1], Yi-Dan Chen [1], Jun-Ping Shi[1], Jin Yang[1,*]

[1] Center for Translational Medicine, The Affiliated Hospital of Hangzhou Normal University, Hangzhou, Zhejiang, China.

[2] Department of occupational medicine, Hangzhou Red Cross Hospital, Hangzhou, Zhejiang, China.

Correspondence should be addressed to Jin Yang, The Affiliated Hospital of Hangzhou Normal University, No. 126, Wenzhou Road, Hangzhou, China. Tel.: 086-571-88358063; E-mail address: hz_zhiy@163.com.

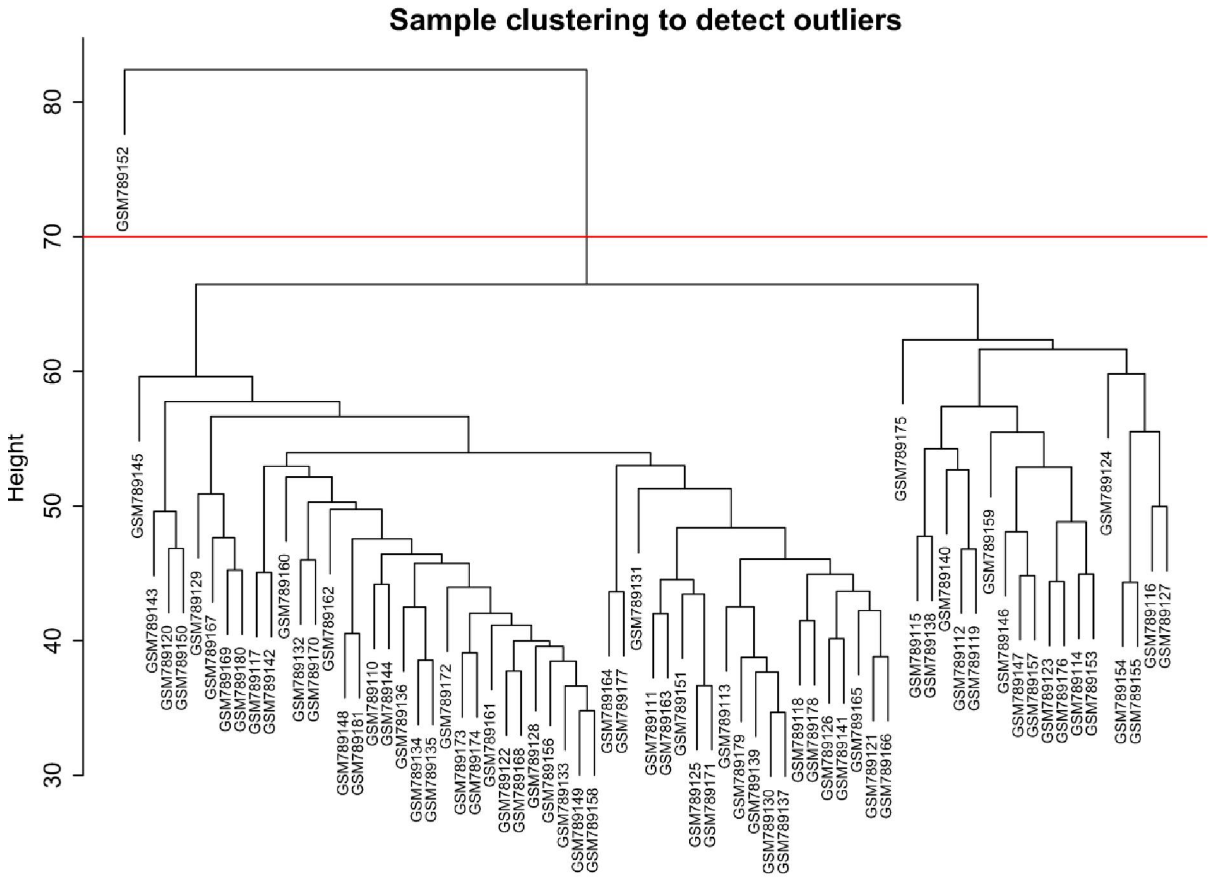

Supplementary Fig. S1. Hierarchical average linkage clustering to detect outlier samples.

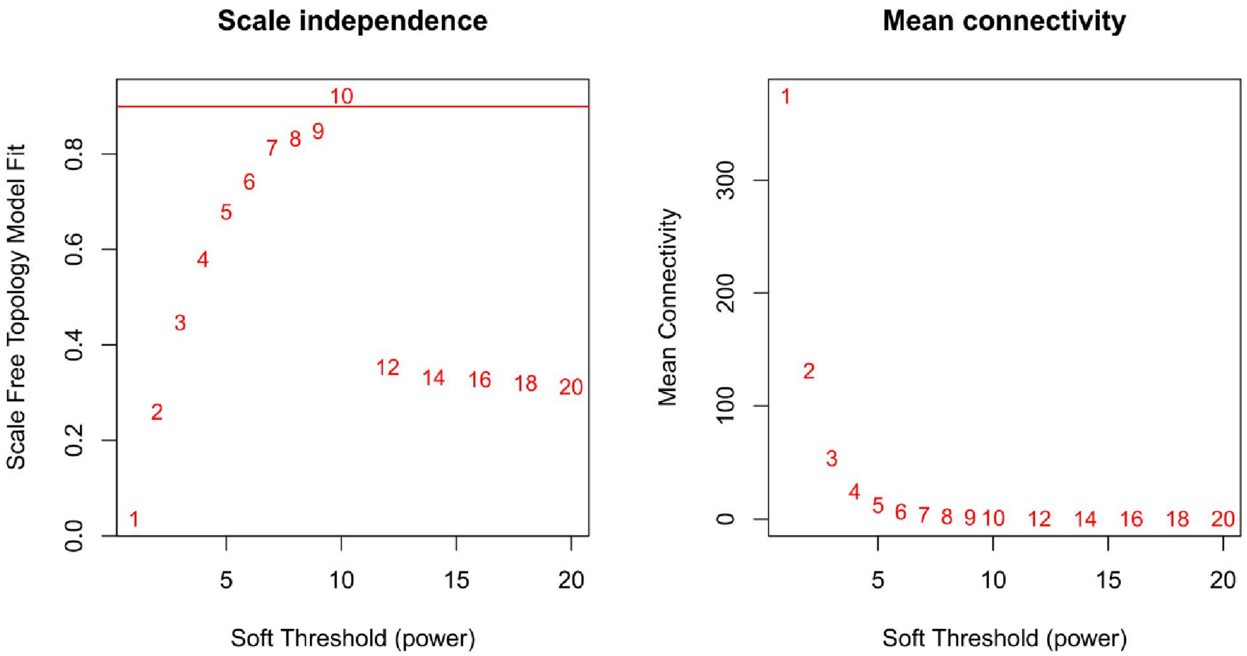

Supplementary Fig. S2. Scale-free feature of the network using DEGs.

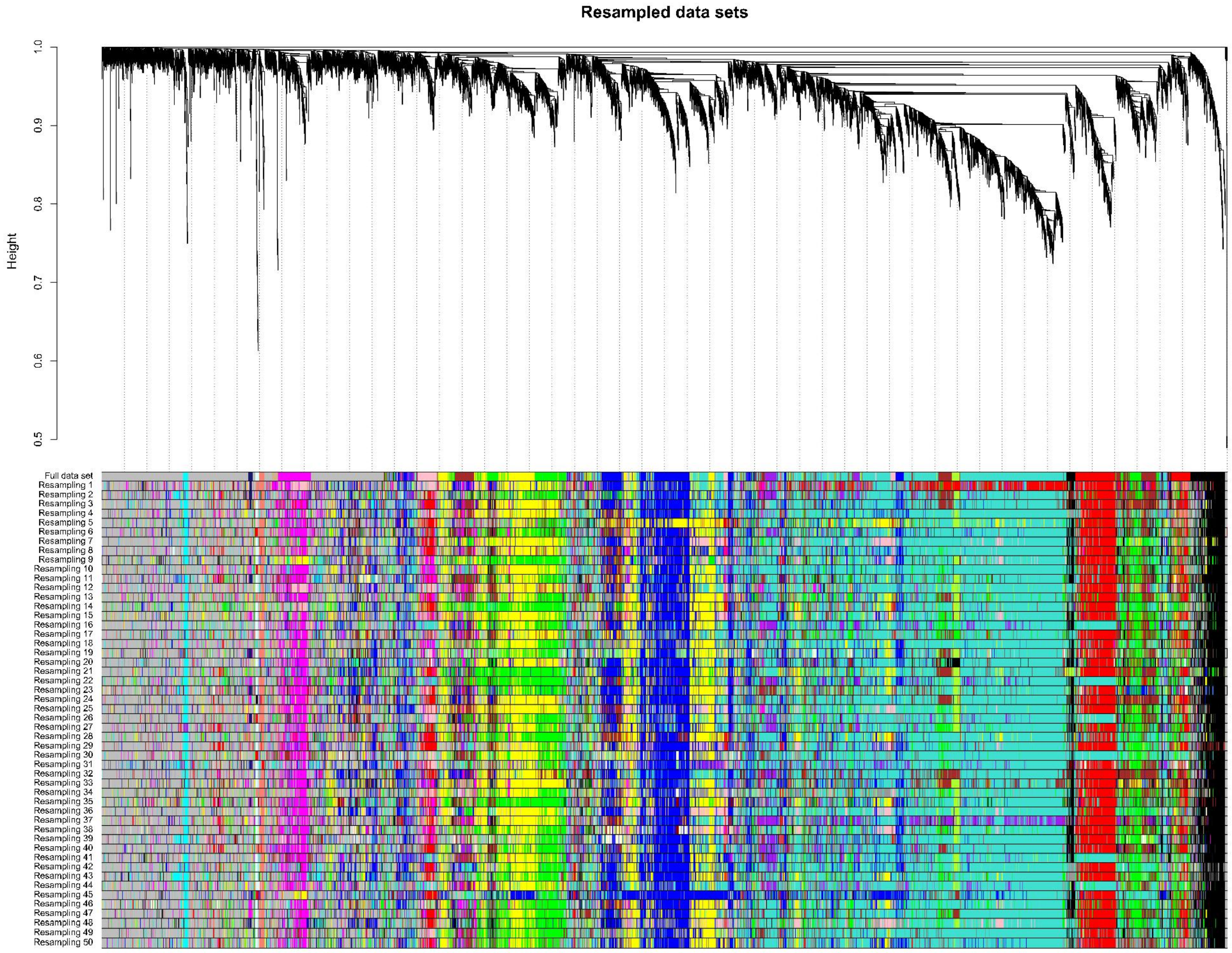

Supplementary Fig. S3. Resampling for module assignments. Module stability study using resampling of samples. The upper panel shows the hierarchical clustering dendrogram of all genes. Branches of the dendrogram correspond to modules, identified by solid blocks of colors in the color row labeled "Full data set". Color rows beneath the first row indicate module assignments obtained from networks based on resampled sets of samples.

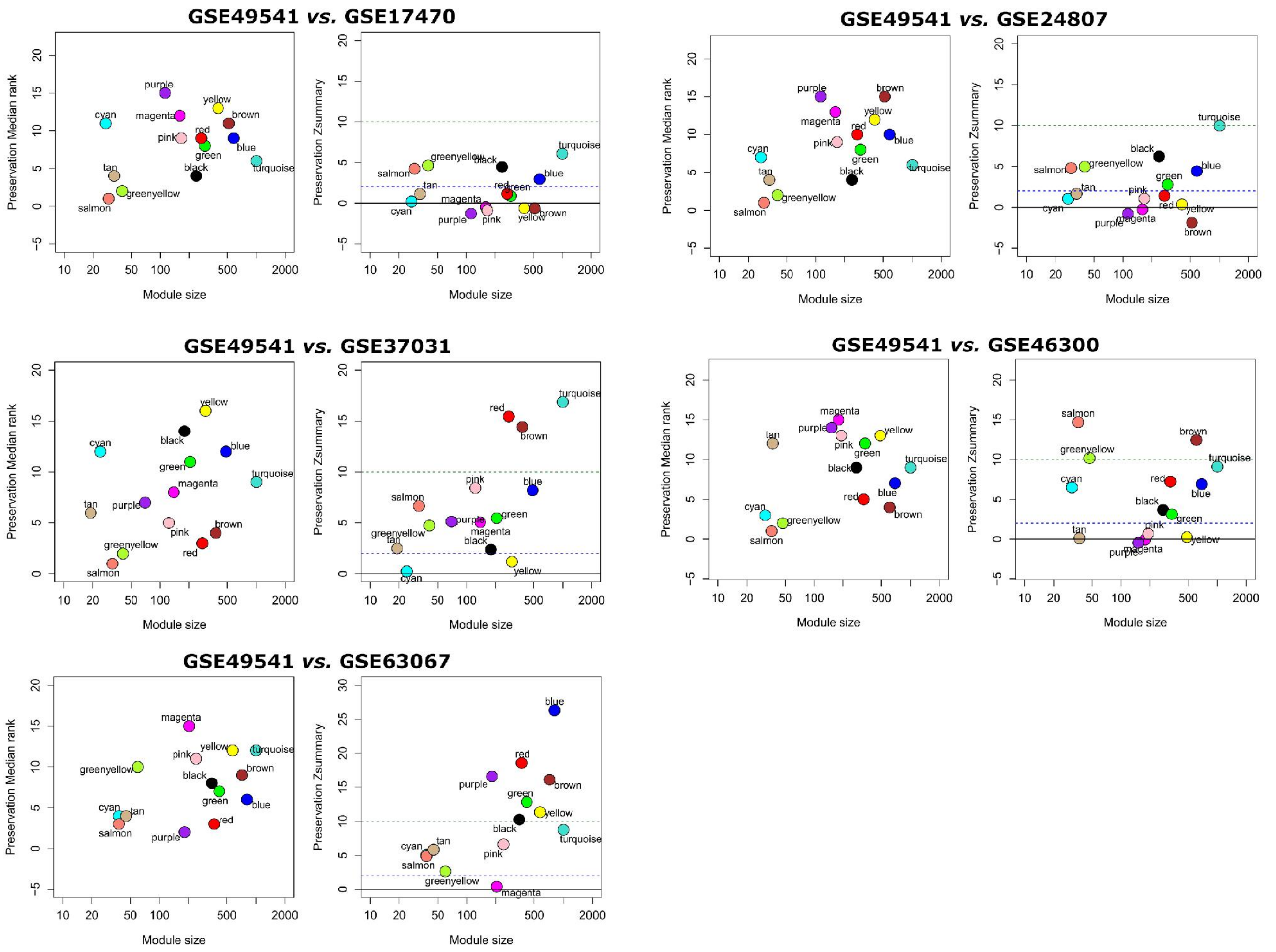

Supplementary Fig. S4. Preservation of GSE49541 network modules in other NAFLD datasets. Each module is represented by its color-code and name. Left figure shows the composite statistic Preservation median rank. This measure tends to be independent from module size with high median ranks indicating low preservation. Right figure shows Preservation Zsummary statistic. The dashed blue (low) and green (high) lines are thresholds highlighting 2 < Z <10 region. This measure is size dependent with Z < 2 indicating low preservation and Z > 10 implying high preserved modules. NAFLD modules (blue, black) show high preservation statistics across all the datasets.

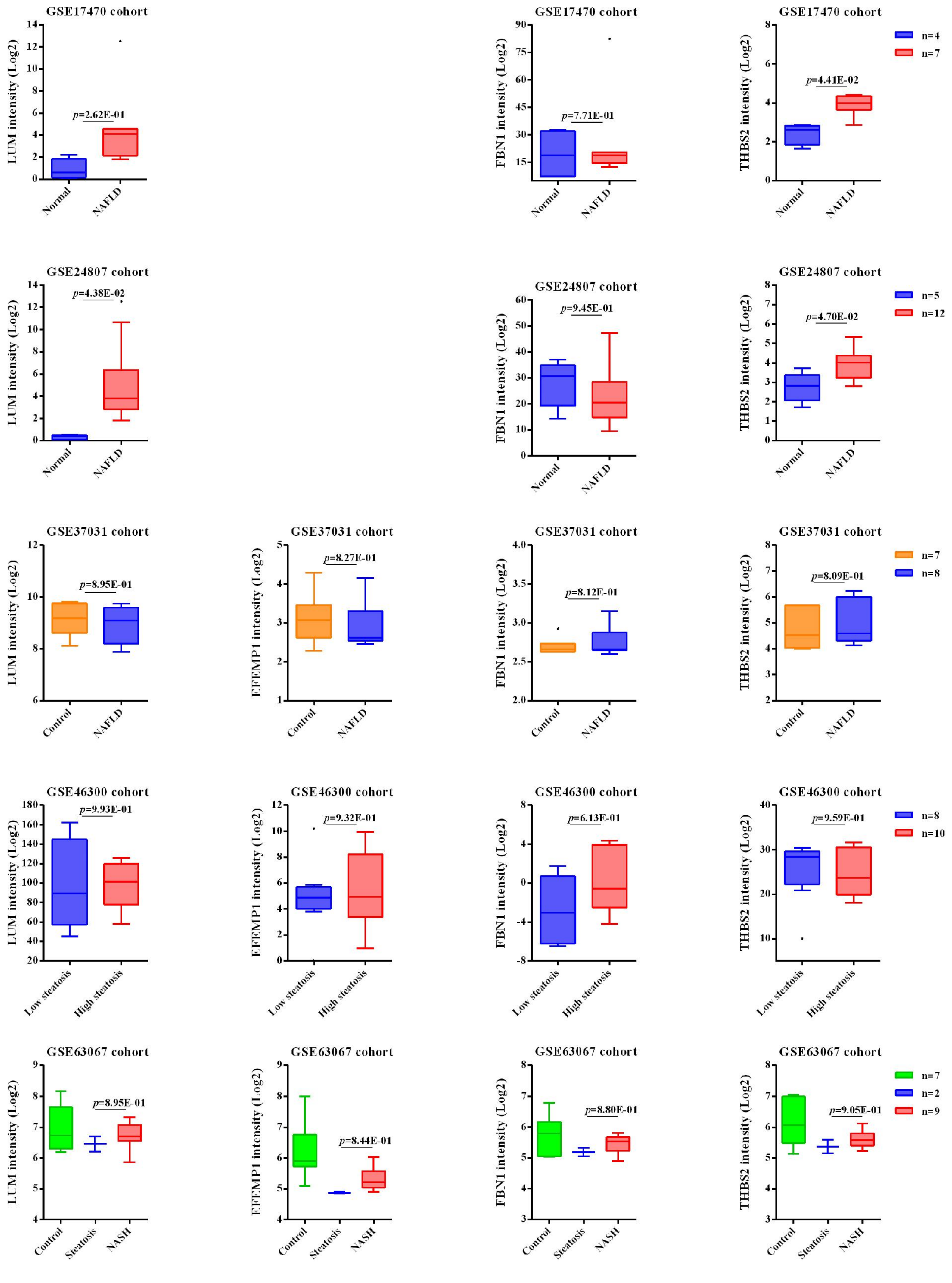

Supplementary Fig. S5. Different datasets were used to evaluate expression of LUM, EFEMP1, FBN1, and THBS2 in liver tissues from NAFLD patients or controls. GSE17470 and GSE24807

used CodeLink Human Whole Genome Bioarray (GE, 2005), which does not contain the probes mapping to EFEMP1. Data were shown as box and whisker plot. Four fibrosis related genes (LUM, EFEMP1, FBN1, and THBS2) were not differential expressed in fibrosis-free NAFLD datasets.

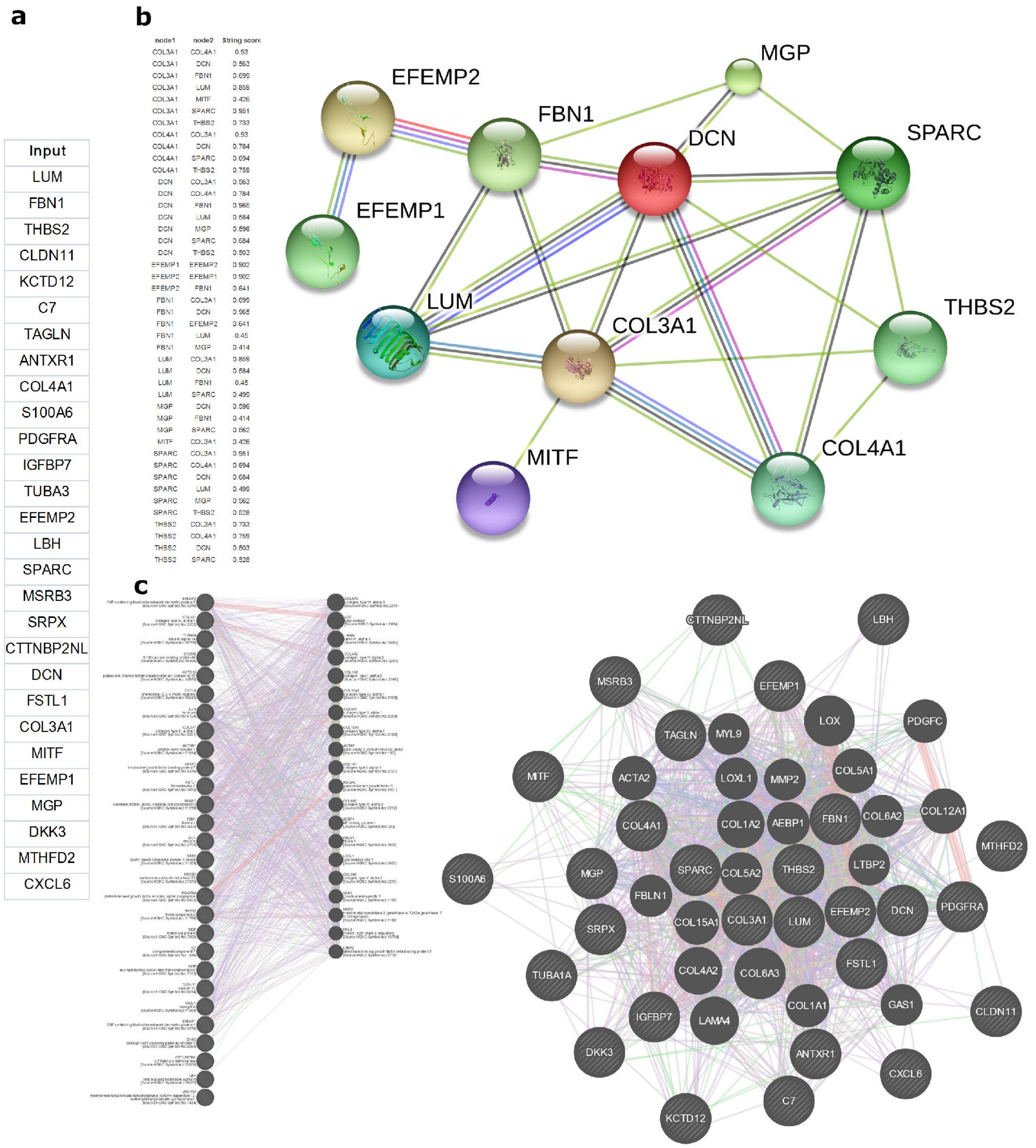

Supplementary Fig. S6. Key genes in module magenta.

(a). To present the key genes of module magenta clearly, edges with TOM ≥ 0.1 (28 nodes, 61edges) were imported into Cytoscape to present the co-expression network, as shown in Fig.5b. Here, these 28 genes were also used to construct the network by different algorithm. (b). Network prepared by String database [1]. Edge scores were also shown. (c). Network prepared by Genemania [2]. Both String and Genemania integrate physical interactions and functional interactions. Topology of Key genes including 4 hub genes were well preserved among the networks produced by different methods.

LINCS_L1000_Chem_Pert_up	P-value	Adjusted P-value	Z-score	Combined Score
LJP006_HME1_24H-afatinib-0.12	1.62E-14	3.40E-10	-1.79	38.94
LJP005_HS578T_24H-GSK-690693-0.37	4.26E-13	2.99E-09	-1.85	36.41
LJP006_HME1_24H-gefitinib-0.37	1.84E-13	1.93E-09	-1.81	36.38
LJP009_HEPG2_24H-JNK-IN-5A-10	6.96E-13	3.66E-09	-1.86	36.17
LJP006_HME1_24H-selumetinib-10	9.35E-13	3.94E-09	-1.85	35.90
LJP006_BT20_3H-THZ-2-98-01-3.33	3.90E-12	1.03E-08	-1.95	35.83
LJP006_MDAMB231_3H-CGP-60474-0.37	2.14E-11	3.21E-08	-2.02	34.86
LJP006_HME1_24H-ZSTK-474-0.12	2.38E-11	3.35E-08	-2.01	34.52
LJP005_HS578T_3H-GDC-0980-0.12	1.12E-10	1.24E-07	-2.08	33.08
LJP006_HME1_24H-PD-0325901-0.12	6.65E-12	1.56E-08	-1.84	33.01

LINCS_L1000_Chem_Pert_down	P-value	Adjusted P-value	Z-score	Combined Score
LJP006_A549_24H-afatinib-10	3.42E-18	6.74E-14	-1.94	58.92
LJP006_HME1_24H-KIN001-043-1.11	5.27E-16	3.47E-12	-1.96	51.69
LJP006_HCC515_24H-afatinib-10	4.77E-16	3.47E-12	-1.93	50.99
LJP006_HME1_24H-sirolimus-10	3.90E-14	5.58E-11	-2.15	50.87
LJP006_A375_24H-KIN001-043-3.33	2.43E-15	1.20E-11	-2.00	50.22
LJP006_SKBR3_24H-JW-7-24-1-10	3.96E-14	5.58E-11	-2.05	48.32
LJP005_A549_24H-ruxolitinib-10	1.73E-12	1.07E-09	-2.33	48.22
LJP006_HEPG2_24H-GSK-2126458-3.33	1.34E-14	3.31E-11	-1.99	48.01
LJP006_A375_24H-QL-X-138-10	2.98E-14	4.90E-11	-1.99	47.25
LJP006_HME1_24H-sorafenib-10	8.30E-14	1.02E-10	-2.04	46.86

HMDB_Metabolites	P-value	Adjusted P-value	Z-score	Combined Score
Famotidine (HMDB01919)	8.68E-07	2.12E-04	-0.68	5.76
L-Glutamic acid (HMDB00148)	5.38E-05	6.56E-03	-0.80	4.01
C47H89O19P3 (HMDB10120)	5.88E-01	6.21E-01	-1.10	0.53
C47H83O19P3 (HMDB10049)	5.88E-01	6.21E-01	-1.10	0.53
C47H83O19P3 (HMDB10050)	5.88E-01	6.21E-01	-1.10	0.53
C43H83O19P3 (HMDB10037)	5.88E-01	6.21E-01	-1.10	0.53
C47H89O19P3 (HMDB10103)	5.88E-01	6.21E-01	-1.10	0.53
C43H81O19P3 (HMDB10073)	5.88E-01	6.21E-01	-1.10	0.52
C45H85O19P3 (HMDB10040)	5.88E-01	6.21E-01	-1.10	0.52
C45H87O19P3 (HMDB10115)	5.88E-01	6.21E-01	-1.10	0.52

Supplementary Fig. S7. Enrichment analysis of module magenta against ChEA database.

Supplementary Table.S1 Datasets used in this study

Accession	Platform	Title	Samples	Brief information
GSE49541	GPL570: Affymetrix Human Genome U133 Plus 2.0 Array	Expression data for Nonalcoholic fatty liver disease patients	72	40 with mild NAFLD, fibrosis stage 0-1; 32 with advanced NAFLD, fibrosis stage 3-4
GSE48452	GPL11532: Affymetrix Human Gene 1.1 ST Array [transcript (gene) version]	Human liver biopsy of different phases from control to NASH	73	73 samples of human liver grouped into C (control=14), H (healthy obese=27), S (steatosis=14) and N (nash=18).
E-MEXP-3291	A-AFFY-183: Affymetrix GeneChip Human Gene 1.0 ST Array [HuGene-1_0-st-v1](Release 29)	Transcription profiling by array of H. sapiens liver cells to investigate global gene expression associated with the progression of NAFLD	45	The samples were diagnosed as normal (*n* = 19), steatotic (*n* = 10), NASH with fatty liver (*n* = 9), and NASH without fatty liver (*n* = 7).
GSE63067	GPL570:[HG-U133_Plus_2] Affymetrix Human Genome U133 Plus 2.0 Array	Expression data from human non-alcoholic fatty liver disease stages	18	2 human steatosis and 9 human non-alcoholic steatohepatitis (NASH) together with their respective control patterns
GSE59045	GPL15207:[PrimeView] Affymetrix Human Gene Expression Array	Gene expression in liver of morbidly obese patients	15	group I (<5% steatosis), group II (NAFLD, 30-50% steatosis) and group III (NASH). The 15 samples were used for microarray (nr patients respectively for stages I-II-III: 6-4-5).
GSE46300	GPL10558 Illumina HumanHT-12 V4.0 expression beadchip	Classifying distinct grades of human non-alcoholic fatty liver disease employing a systems biology approach	18	nine obese patients with distinct grades of steatosis.
GSE17470	GPL2895 GE Healthcare/Amersham Biosciences CodeLink Human Whole Genome Bioarray	Global gene expression of NASH patients	11	Four normal control ,six NASH patients exhibiting significant insulin resistence were included in this study.
GSE24807	GPL2895 GE Healthcare/Amersham Biosciences CodeLink Human Whole Genome Bioarray	Increased hemoglobin expression in NASH livers	17	Twelve biopsy diagnosed NASH patients were included in this study. Five normal control.
GSE37031	GPL14877 Affymetrix Human Genome U133 Plus 2.0 Array [Brainarray Version 13, HGU133Plus2_Hs_ENTR	Transcriptome Analysis from non-alcoholic steatohepatitis (NASH)	15	8 non-alcoholic steatohepatitis (NASH) and 7 control samples.

	EZG]			
GSE84044	GPL570 [HG-U133_Plus_2] Affymetrix Human Genome U133 Plus 2.0 Array	Characterization of gene expression profile in HBV-related liver fibrosis patients	124	124 chronic hepatitis B (CHB) patients. The pathological Scheuer Score of each sample were evaluated base on the inflamation and fibrosis severity.

Supplementary Table.S2 Primers used in this study

Gene Symbol	GeneID		Sequence	Tm
LUM	4060	Forward	CTGCGTTTATCTCACAACGAACT	61.1
		Reverse	CAGATCCAGCTCAACCAGGG	62
EFEMP1	2202	Forward	GTCACAGGACACCGAAGAAAC	60.8
		Reverse	TTGCATTGCTGTCTCACAGGA	62
FBN1	2200	Forward	CAGGACAGGCCCATGTTTTAC	60.9
		Reverse	GCACAGCAGAGCGTTTTTGT	62
THBS2	7058	Forward	ATAGACAGCTTCGCTCTGGAC	61.3
		Reverse	CAAACCCCTGAAGTGACTCTC	60
GAPDH	2597	Forward	ACAACTTTGGTATCGTGGAAGG	60.2
		Reverse	GCCATCACGCCACAGTTTC	61.7
Lum	17022	Forward	CTCTTGCCTTGGCATTAGTCG	61
		Reverse	GGGGGCAGTTACATTCTGGTG	62.7
Efemp1	216616	Forward	GCGCTGGTCAAGTCACAGTA	62.1
		Reverse	AAGCATCTGGGACAATGTCAC	60.3
Fbn1	14118	Forward	GGACGCCAATTTGGAGGCT	62.7
		Reverse	CTTTCAGCGCATCGTGTCCT	62.8
Thbs2	21826	Forward	CTGGGCATAGGGCCAAGAG	61.8
		Reverse	GTCTTCCGGTTAATGTTGCTGAT	60.9
Gapdh	14433	Forward	AGGTCGGTGTGAACGGATTTG	62.6
		Reverse	TGTAGACCATGTAGTTGAGGTCA	60.2

Reference

1 Szklarczyk, D. *et al.* STRING v10: protein-protein interaction networks, integrated over the tree of life. *Nucleic acids research* **43**, D447-452, doi:10.1093/nar/gku1003 (2015).

2 Montojo, J., Zuberi, K., Rodriguez, H., Bader, G. D. & Morris, Q. GeneMANIA: Fast gene network construction and function prediction for Cytoscape. *F1000Research* **3**, 153, doi:10.12688/f1000research.4572.1 (2014).
